# Impact of Seropositivity on Mortality and Extra-Articular Manifestations in Rheumatoid Arthritis: A Nationwide Propensity-Matched Cohort Study

**DOI:** 10.31138/mjr.150425.smr

**Published:** 2025-12-31

**Authors:** Ahmad Alomari, Qusai Alqudah, Geran Maule, Aseed Mestarihi, Samah Al-Omari, Osama Obeidat, Reem Elmusa, Abdallah Rayyan, Omar Obeidat, Safwan Alomari, Diala Alawneh

**Affiliations:** 1 University of Central Florida College of Medicine, Graduate Medical Education, Orlando, FL, USA;; 2 HCA Florida North Florida Hospital, Internal Medicine Residency Program, Gainesville, FL, USA;; 3 Department of Internal Medicine, Jordan University of Science and Technology, Irbid, Jordan;; 4 Johns Hopkins University School of Medicine, Baltimore, Maryland, USA;; 5 Division of Rheumatology, Department of Medicine, University of Florida, Gainesville, Florida, USA

**Keywords:** autoantibodies, disease progression, interstitial lung disease, prognosis, rheumatoid arthritis, serologic tests

## Abstract

**Background/Objective.:**

Rheumatoid arthritis (RA) is a chronic autoimmune disease that manifests as either seropositive or seronegative subtypes. Seropositive RA is often linked to more severe joint damage and systemic complications. In contrast, seronegative RA has a less defined clinical profile but may still present with significant comorbidities. This study aims to compare clinical outcomes between these RA subtypes using real-world data from the TriNetX Research Network.

**Methods.:**

A retrospective cohort study analysed adult RA patients from 2015 to 2025, categorised as seropositive or seronegative using International Classification of Diseases, 10th Revision (ICD-10 codes). The primary outcome was all-cause mortality, while secondary outcomes included hospitalization, steroid dependence, disease-modifying antirheumatic drug (DMARD) use, RA-related joint damage, interstitial lung disease (ILD), and coronary artery disease (CAD).

**Results.:**

Among 106,492 matched patients (53,246 per cohort), seropositive RA patients had higher all-cause mortality (OR: 1.241; p < 0.001) and increased risks of DMARD use, steroid dependence, and joint damage. They also showed a greater incidence of ILD (OR: 2.419; p < 0.001), CAD indicating a more severe disease course.

**Conclusion.:**

This study highlights significant differences in several clinical outcomes between seropositive and seronegative RA patients. These findings highlight the more aggressive nature of seropositive disease and its extra-articular involvement and reinforce the importance of autoantibody status in prognostication and risk stratification for RA patients.

## INTRODUCTION

Rheumatoid arthritis (RA) is a chronic, systemic auto-immune disease characterised by persistent synovial inflammation, progressive joint destruction, and significant extra-articular manifestations. Affecting approximately 0.5–1% of the global population, RA remains a leading cause of disability, particularly among women, who are disproportionately affected. Furthermore, RA is associated with an increased risk of mortality compared to the general population.^1,2^ The disease is broadly categorised into seropositive and seronegative RA based on the presence or absence of rheumatoid factor (RF) and anti-citrullinated peptide antibodies (ACPA).

The genetic basis of RA varies between seropositive and seronegative disease. Seropositive RA is strongly associated with HLA-DRB1 alleles, particularly the shared epitope hypothesis, which influences autoantigen presentation and immune activation.^3,4^ In contrast, other genetic factors have been linked to ACPA-negative disease,^5^ suggesting that these subsets may represent partly distinct disease entities. Pathophysio-logically, seropositive RA is characterised by immune complex formation involving RF and ACPA, leading to chronic synovitis, erosive joint disease, and extra-articular manifestations. Seronegative RA, on the other hand, is often considered a more heterogeneous entity with varying inflammatory pathways, potentially involving IL-6, TNF-alpha, and non-ACPA-related mechanisms.

Seropositive RA, which accounts for 60–80% of RA cases, is associated with a stronger autoantibody-mediated immune response. This results in greater synovial inflammation, more aggressive joint damage, and higher extra-articular involvement, including interstitial lung disease (ILD) and RA- related lung disease, cardiovascular disease (CVD), and rheumatoid vasculitis.^6,7^ Conversely, seronegative RA has traditionally been perceived as a milder form of the disease. However, recent studies challenge this assumption, indicating that seronegative RA patients may experience delayed diagnosis due to the absence of serological markers, which may lead to treatment initiation at a more advanced disease stage.^8^

Given the differences in disease trajectory, systemic involvement, and potential variations in treatment efficacy between seropositive and seronegative RA, it is crucial to assess long-term outcomes in both patient populations. While previous studies have examined disease progression and treatment responses, large-scale real-world data are needed to validate these findings across diverse populations. This study leverages TriNetX, a global federated health research network, to conduct a retrospective cohort analysis comparing key outcomes in seropositive and seronegative RA patients. The study population is derived from electronic medical records of over 100 healthcare organisations, ensuring a large and diverse dataset.

## METHODS

### Study Oversight and Data Source

This study used deidentified data from the TriNetX Research Network, a federated database that aggregates de-identified electronic health records (EHRs) from more than 120 healthcare organisations and over 250 million patients globally. For this analysis, the U.S. Collaborative Network within TriNetX was used, comprising data from 103 healthcare organisations. TriNetX ensures data quality through standardised metrics for conformance, completeness, and plausibility. We defined the study period from February 1, 2015, to February 1, 2025. Data extraction and analysis were completed on Feb 14, 2025. TriNetX Network is compliant with the Health Insurance Portability and Accountability Act (HIPAA) and the General Data Protection Regulation (GDPR).

### Study Population

The study included adult patients aged 18 years or older who were diagnosed with rheumatoid arthritis identified using the International Classification of Diseases, 10th Revision (ICD-10) codes. Patients were further classified into two cohorts: Seropositive and Seronegative RA. The Seropositive RA cohort was defined using ICD-10 code M05 (Rheumatoid arthritis with rheumatoid factor) and included patients who either had this diagnosis alone or in combination with CPT code 86200 (Cyclic citrullinated peptide [CCP], antibody). The Seronegative RA cohort was defined using ICD-10 code M06.0 (Rheumatoid arthritis without rheumatoid factor) and excluded patients with M05 or CPT code 86200. The Supplemental Appendix describes the Current Procedural Terminology and ICD-10 codes used for cohort identification, study window definitions, and Propensity Score Matching (PSM) characteristics.

### Study Outcome

The outcomes of interest were all-cause mortality, hospitalisation, development of ILD and RA- related lung disease, pulmonary hypertension (PH), steroid dependence, disease modifying antirheumatic drug (DMARD) use, RA-related joint damage, and coronary artery disease. Index date was defined as the date of the first recorded seropositive or seronegative RA diagnosis. Outcomes were assessed from the index date and monitored throughout the study period which was 10 years (3,650 days). To ensure accurate analysis, all outcomes were identified using standardised diagnostic and procedural codes available in the TriNetX database.

### Statistical Analysis

Baseline characteristics of the two cohorts were summarised using descriptive statistics. Continuous variables were reported as means and standard deviations and compared using t-tests. Categorical variables were expressed as counts and percentages and compared using chi-square tests. PSM was conducted using Tri-NetX’s built-in algorithm, which employs a greedy nearest-neighbour approach with a caliper of 0.1 pooled standard deviations. Matching balance was assessed using standardised mean differences, with values less than 0.1 indicating acceptable balance between the seropositive and seronegative rheumatoid arthritis cohorts. The characteristics on which we performed PSM are included in the Appendix.

The incidence of outcomes was compared between cohorts using risk ratios, odds ratios, and risk differences, all with 95% confidence intervals. Time-to-event outcomes were analysed using Kaplan-Meier survival curves, with differences assessed using log-rank tests. Hazard ratios and their 95% confidence intervals were calculated using Cox proportional hazards models. All statistical analyses were performed using the TriNetX platform, which integrates these methodologies into its built-in analytical tools. A two-tailed p-value of <0.05 was considered statistically significant. The integrated R Survival package was used for the analysis on the TriNetX platform.

To account for the risk of type I error due to multiple outcome testing, we interpreted p-values in the context of clinical relevance and consistency with prior literature. Formal adjustments such as Bonferroni correction were not applied due to the exploratory nature of this real-world cohort study, but major findings were supported by strong statistical significance (p < 0.001) and effect sizes.

## RESULTS

### Characteristics of Study Participants

**Figure 1.**
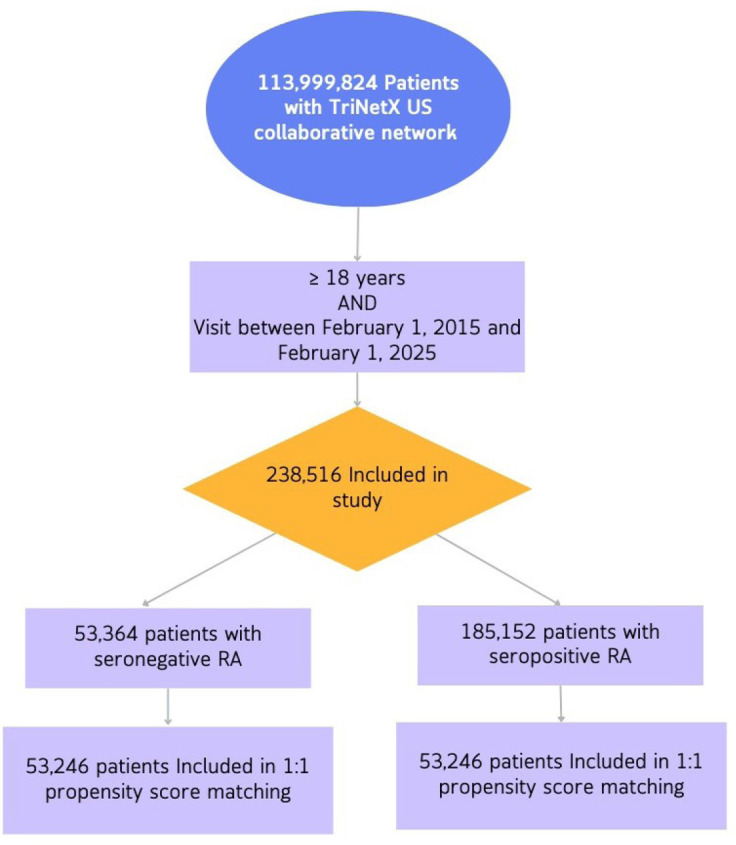
Flowchart of the selection process.

This retrospective study identified 238,517 patients diagnosed with rheumatoid arthritis, of whom 185,153 were classified as seropositive and 53,364 as seronegative. After applying PSM, the final matched cohort consisted of 53,246 patients in each group, ensuring comparability in demographic and clinical characteristics (**Figure 1**). Prior to matching, significant differences in demographics, comorbid conditions, and medication use were observed. However, after PSM, these differences were largely balanced with age at index. The majority of patients were female, and racial distribution was similar, with 68.4% of the seropositive group and 68.0% of the seronegative group identifying as White. **Table 1** presents the baseline characteristics of the study population before and after matching.

**Table 1.** Baseline characteristics of the patient cohort before and after PSM.

	Before PSM			After PSM		
	Seropositive RA (n = 185,153)	Seronegative RA (n = 53,364)	Standardised Difference	Seropositive RA (n = 53,246)	Seronegative RA (n = 53,246)	Standardised Difference

Demographics						
Age at Index	59.9 ± 14.8	59.3 ± 15.5	0.039	59.4 ± 15.2	59.4 ± 15.5	0.001
Female	135,012 (72.9%)	37,880 (71.0%)	0.043	37,778 (70.9%)	37,803 (71.0%)	0.001
White	113,972 (61.6%)	36,319 (68.1%)	0.136	36,409 (68.4%)	36,225 (68.0%)	0.007
Black or African American	22,181 (12.0%)	4,820 (9.0%)	0.096	4,694 (8.8%)	4,815 (9.0%)	0.008
Comorbid conditions						
Disorders of lipoprotein metabolism and other lipidemias	63,696 (34.4%)	20,406 (38.2%)	0.080	19,932 (37.4%)	20,343 (38.2%)	0.016
Essential (primary) hypertension	74,596 (40.3%)	22,676 (42.5%)	0.045	22,028 (41.4%)	22,612 (42.5%)	0.022
Tobacco use	6,871 (3.7%)	1,477 (2.8%)	0.053	1,409 (2.6%)	1,474 (2.8%)	0.008
Overweight and obesity	31,995 (17.3%)	11,115 (20.8%)	0.090	10,863 (20.4%)	11,057 (20.8%)	0.009
Chronic kidney disease (CKD)	14,842 (8.0%)	4,626 (8.7%)	0.024	4,518 (8.5%)	4,613 (8.7%)	0.006
Type 2 diabetes mellitus	29,419 (15.9%)	8,910 (16.7%)	0.022	8,575 (16.1%)	8,885 (16.7%)	0.016
Arthropathic psoriasis	3,012 (1.6%)	1,671 (3.1%)	0.099	1,606 (3.0%)	1,629 (3.1%)	0.003
Enteropathic arthropathies	162 (0.1%)	192 (0.4%)	0.058	142 (0.3%)	160 (0.3%)	0.006
Ankylosing spondylitis	1,915 (1.0%)	1,232 (2.3%)	0.100	1,157 (2.2%)	1,181 (2.2%)	0.003
Juvenile arthritis	2,700 (1.5%)	774 (1.5%)	0.001	758 (1.4%)	771 (1.4%)	0.002
Systemic lupus erythematosus (SLE)	7,113 (3.8%)	2,310 (4.3%)	0.025	2,210 (4.2%)	2,300 (4.3%)	0.008
Medication						
Beta Blocking Agents	42,716 (23.1%)	13,618 (25.5%)	0.057	13,134 (24.7%)	13,566 (25.5%)	0.019
Angiotensin II Inhibitor	22,357 (12.1%)	7,077 (13.3%)	0.036	6,802 (12.8%)	7,060 (13.3%)	0.014
ACE Inhibitors	27,700 (15.0%)	8,004 (15.0%)	0.001	7,650 (14.4%)	7,981 (15.0%)	0.018
Calcium Channel Blockers	31,123 (16.8%)	9,666 (18.1%)	0.034	9,271 (17.4%)	9,636 (18.1%)	0.018
Loop Diuretics	19,974 (10.8%)	5,891 (11.0%)	0.008	5,704 (10.7%)	5,881 (11.0%)	0.011
Thiazides/Related Diuretics	27,435 (14.8%)	8,591 (16.1%)	0.035	8,211 (15.4%)	8,562 (16.1%)	0.018
Spironolactone	5,558 (3.0%)	1,871 (3.5%)	0.028	1,815 (3.4%)	1,863 (3.5%)	0.005
Aspirin	34,537 (18.7%)	10,857 (20.3%)	0.043	10,459 (19.6%)	10,819 (20.3%)	0.017
Clopidogrel	6,953 (3.8%)	1,985 (3.7%)	0.002	1,958 (3.7%)	1,979 (3.7%)	0.002
Ticagrelor	896 (0.5%)	300 (0.6%)	0.011	282 (0.5%)	300 (0.6%)	0.005
Prasugrel	360 (0.2%)	110 (0.2%)	0.003	101 (0.2%)	110 (0.2%)	0.004
Lipid Modifying Agents	47,293 (25.5%)	14,801 (27.7%)	0.050	14,351 (27.0%)	14,764 (27.7%)	0.017
Blood Glucose Lowering Drugs, excl. Insulins	18,667 (10.1%)	5,939 (11.1%)	0.034	5,700 (10.7%)	5,919 (11.1%)	0.013
Insulin	15,347 (8.3%)	4,729 (8.9%)	0.020	4,548 (8.5%)	4,712 (8.8%)	0.011
Antithyroid Agents	764 (0.4%)	197 (0.4%)	0.007	182 (0.3%)	196 (0.4%)	0.004

Values are mean ± SD or n (%).

CKD: Chronic kidney disease; SLE: Systemic lupus erythematosus; ACE: angiotensin-converting enzyme.

### Outcomes

**Figure 2.**
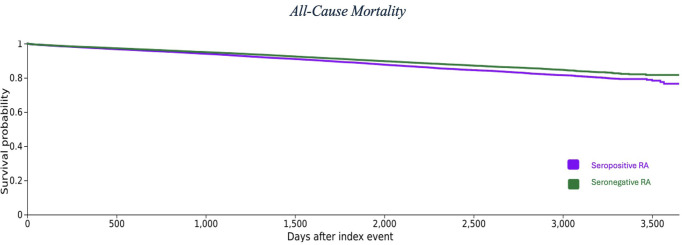
All-cause mortality.

The primary outcome of all-cause mortality was higher in the seropositive RA group compared to the seronegative group (odds ratio [OR]: 1.241; 95% confidence interval [CI]: 1.184–1.302; p < 0.001). Kaplan-Meier analysis demonstrated lower survival probabilities in the seropositive group (76.51% vs. 81.68%; log-rank p < 0.001), with a hazard ratio of 1.210 (95% CI: 1.156–1.266), indicating a significantly increased risk (**Figure 2**).

Hospitalisation rates were slightly higher among seropositive patients (OR: 1.028; 95% CI: 1.003–1.053; p = 0.028). The incidence of ILD and RA- related lung disease was significantly higher in the seropositive group (OR: 2.419; 95% CI: 2.262–2.587; p < 0.001). Similarly, PH was more prevalent in the seropositive group (OR: 1.338; 95% CI: 1.246–1.436; p < 0.001).

Moreover, there was a statistically significant difference, with steroid dependence being more common in the seropositive group (OR: 1.178; 95% CI: 1.134–1.224; p < 0.001), as was the use of disease-modifying antirheumatic drugs (OR: 1.347; 95% CI: 1.305–1.390; p < 0.001).

A few other complications were also more prevalent among seropositive RA patients. For example, the risk of RA-related joint damage was significantly higher in seropositive patients (OR: 1.236; 95% CI: 1.148–1.332; p < 0.001). Similarly, chronic CAD was more frequently observed (OR: 1.106; 95% CI: 1.058–1.157; p < 0.001), as was acute coronary syndrome (OR: 1.096; 95% CI: 1.024–1.172; p = 0.008). Kaplan-Meier analyses for several of these conditions consistently demonstrated worse survival outcomes for seropositive RA patients. **Table 2** summarises the outcomes of the study.

**Table 2.** Outcomes.

Outcome	n (%)	n (%)	Odds Ratio (95% CI)	P Value
All-Cause Mortality “The primary outcome”	4,093 (7.7%)	3,348 (6.3%)	1.241 (1.184–1.302)	< 0.001
Hospitalisation	22,253 (41.8%)	21,900 (41.1%)	1.028 (1.003–1.053)	0.028
Interstitial lung disease and RA- related lung disease	2,908 (5.7%)	1,268 (2.4%)	2.419 (2.262, 2.587)	< 0.001
Pulmonary Hypertension	1,817 (3.5%)	1,375 (2.6%)	1.338 (1.246–1.436)	< 0.001
Steroid Dependence	6,303 (11.8%)	5,448 (10.2%)	1.178 (1.134–1.224)	< 0.001
DMARDs Use	14,989 (45.8%)	12,027 (38.6%)	1.347 (1.305–1.390)	< 0.001
RA-Related Joint Damage	1,579 (3.1%)	1,289 (2.5%)	1.236 (1.148–1.332)	< 0.001
Chronic CAD	4,441 (9.3%)	4,059 (8.5%)	1.106 (1.058–1.157)	< 0.001
Acute Coronary Syndrome (ACS)	1,822 (3.5%)	1,670 (3.2%)	1.096 (1.024–1.172)	0.008

Values are n (%).

CI: confidence interval; RA: rheumatoid arthritis; DMARDs: disease-modifying antirheumatic drugs; CAD: coronary artery disease.

## DISCUSSION

In this retrospective cohort analysis of patients with RA, we identified distinct differences in clinical outcomes between seropositive and seronegative RA subtypes. Seropositive RA was associated with increased all-cause mortality, a higher risk of hospitalisation, and a greater burden of extra articular manifestations, including ILD and RA- related lung disease and PH. Additionally, these patients exhibited an elevated risk of both acute and chronic CAD, greater dependence on corticosteroids, higher treatment retention with DMARDs and more severe joint damage. These findings underscore the need for tailored screening and therapeutic strategies to optimise long-term outcomes in this high-risk RA population.

The pathogenesis of RA involves a complex interplay between B cells, T cells, and dendritic cells, with environmental and genetic factors leading to loss of tolerance to citrullinated proteins and the production of autoantibodies like ACPA and RF.^9^ Seropositive RA is associated with increased all-cause mortality compared to seronegative RA. Studies have shown that seropositive patients have a higher risk of death, with mortality rates significantly elevated in those testing positive for RF and ACPA autoantibodies.^10–12^ The underlying mechanisms contributing to this increased mortality may involve more aggressive disease progression and a higher incidence of comorbid conditions, such as cardiovascular and respiratory diseases in seropositive individuals.^13,14^ Our study supports these findings, demonstrating a higher all-cause mortality rate among seropositive RA patients, reinforcing the importance of early and aggressive management in this population. In our study, seropositive RA patients had a significantly higher prevalence of ILD or RA- related lung disease (5.7% vs. 2.4%) with a significant reduction in disease-free survival. The literature suggests that 3–10% of RA patients eventually develop ILD,^15–18^ and RA-ILD is associated with a threefold increase in mortality.^16^ Retrospective analysis has shown that combined RF and ACPA seropositivity increases ILD risk, with higher RF levels strongly associated with ILD development.^17^ A meta-analysis of 17 studies, including 992 RA-ILD and 2,223 RA-non-ILD patients, found that ACPA positivity significantly increased the likelihood of RA-ILD, with higher ACPA titres correlating with greater risk.^19^ Mechanistically, RA-ILD is thought to involve citrullinated peptide-driven lung fibroblast activation, leading to fibrosis, particularly in usual interstitial pneumonia (UIP).^19^

Similarly, PH was more prevalent in seropositive RA patients, with Kaplan-Meier analysis revealing lower survival probabilities in this group. The pathogenesis of PH in RA likely involves vascular inflammation and secondary effects of ILD.^20^ RA-associated PH falls under Group 1 pulmonary arterial hypertension (PAH), which is characterised by pulmonary artery vascular cell proliferation and luminal narrowing.^21^ Studies have reported elevated pulmonary artery pressures (≥30 mm Hg) in 21–27.5% of RA patients, highlighting the need for early screening and intervention.^22,23^

Both acute and chronic CAD were slightly more prevalent in the seropositive RA cohort in our study. Several studies indicate that seropositive RA, particularly in patients with RF and anti-CCP antibodies, is strongly linked to an increased risk of CAD and cardiovascular mortality.^24–26^ Systemic inflammation, an altered lipid profile, and glucocorticoid use contribute to this increased cardiovascular risk.^27–29^ Autoimmune mechanisms also play a key role, as ACPAs and RF have been associated with acute coronary syndrome (ACS), stroke, and major adverse cardiovascular events (MACE).^30^ Additionally, anti-CCP antibodies have been identified as independent risk factors for CAD. Citrullinated proteins are widely present within atherosclerotic plaques and have been linked to a higher atherosclerotic burden. These findings suggest that the immune response targeting citrullinated epitopes, particularly citrullinated fibrinogen, within atherosclerotic plaques may contribute to the accelerated progression of atherosclerosis in RA patients.^31^ Moreover, RA-related autoantibodies have been linked to subclinical atherosclerosis even in non-RA populations, highlighting their broader role in cardiovascular pathology.^32^ Given this strong association, early cardiovascular risk management is essential in RA, particularly for seropositive patients. Autoantibodies in seropositive RA, such as RF and ACPA contribute to a more aggressive disease course through chronic immune activation and sustained inflammation.^33^ This elevated disease activity often necessitates increased use of DMARDs and glucocorticoids to manage inflammation and prevent progression.^34^ ASCORE study has demonstrated that seropositive patients are more likely to be prescribed DMARDs and require glucocorticoid therapy initiation or dose escalation compared to seronegative patients, reflecting a greater need for aggressive treatment strategies.^35^ A secondary analysis of CARDERA trial finds higher Disease Activity Scores (DAS) in seropositive individuals, indicating more severe joint inflammation and overall disease burden.^36^ The presence of ACPA has been linked to increased joint destruction and radiographic progression, further supporting the association between seropositivity and worse clinical outcomes.^37^ Moreover, seropositive patients often exhibit higher levels of systemic inflammation markers, including C-reactive protein (CRP) and erythrocyte sedimentation rate (ESR), correlating with disease severity.^38^ Consistent with these findings, our study also observed higher DMARD use and steroid dependence in the seropositive group, reinforcing the theory that seropositivity is a predictor of more aggressive RA and increased treatment burden. Our findings of increased DMARD use and steroid dependence in seropositive RA are consistent with prior reports indicating that seropositive disease often requires more aggressive and sustained treatment. Notably, a recent nationwide study published in the Mediterranean Journal of Rheumatology reported higher drug retention rates among seropositive RA patients compared to seronegative counterparts, reflecting greater disease persistence and treatment intensity.^39^ This further underscores the clinical severity and therapeutic challenges associated with seropositivity.

Our study’s findings align with existing literature indicating that seropositive RA patients experience more pronounced joint deformities and damage compared to seronegative RA patients. Pathological ultrasound findings have been reported to be more prevalent in seropositive patients, particularly in the 2nd metacarpophalangeal (MCP) joint, with higher occurrences in both grayscale and power Doppler imaging.^39^ Additionally, erosions appear more frequently in seropositive individuals.^39^ Lesions in proximal interphalangeal (PIP), MCP, and wrist joints, along with advanced bone damage, joint subluxations, dislocations, and joint space narrowing, are also more commonly observed in seropositive RA patients.^38^ Furthermore, the progression of damage in seropositive RA has been associated with higher disease activity levels and independent effects of RF, particularly on bone damage, emphasising the importance of considering RF status regardless of disease activity.^37^. These findings provide evidence that seropositive RA is linked to more severe joint pathology and deformities, consistent with our results.

This study builds upon existing literature in the Mediterranean Journal of Rheumatology by offering the first large-scale, propensity-matched cohort comparison of seropositive and seronegative RA using real-world data from over 100 healthcare systems. Whereas prior MJR studies have focused on localised or single-institution experiences, our analysis confirms the prognostic impact of seropositivity and adds new insights into extra-articular complications such as ILD, pulmonary hypertension, and cardiovascular disease. The use of survival analyses and risk-adjusted estimates enhances interpretability, positioning this study as a significant contribution to the journal’s RA portfolio.

### Limitations and Future Directions

One limitation of our study is the observational retrospective design. Selection bias and confounding remain potential concerns. However, a strict propensity score matching was applied to minimise bias and confounding of different variables, yielding similar, matched cohorts, and improving the internal validity of the paper. In addition, our analysis identified significant differences in the outcomes between seropositive and seronegative RA patients. While the differences in several of these outcomes were found to be statistically significant, the magnitude of these differences may not be clinically significant. For example, the odds ratio for difference in hospitalisation rate for seropositive to seronegative RA patients was 1.028 [CI: 1.003 -1.053], P = 0.28. This difference can be explained by the prior literature finding that seropositive patients tend to have more symptomatic and severe disease compared to seronegative patients, which can affect the outcomes.^33^ On the other hand, the reason behind the statistical significance of these differences might be the relatively large sample size in our study. Therefore, these factors should be taken into consideration by the readership when interpreting the results of our study and future prospective studies can further confirm or negate these findings.

Although we performed extensive propensity score matching using 49 variables—including demographics, medications, and comorbid conditions such as SLE, psoriasis, and ankylosing spondylitis—unmeasured confounding from other CTD overlap syndromes (e.g., mixed connective tissue disease or undiagnosed myositis) cannot be completely excluded. These conditions may independently influence the risk of outcomes like ILD and warrant consideration in future prospective studies.

Another limitation is the reliance on ICD-10 coding, which can introduce misclassification bias. The accuracy of coding may vary depending on whether entries are made by clinicians or non-clinical personnel, potentially impacting cohort identification and outcome capture.

## Data Availability

The data supporting the findings of this study are available from the TriNetX Research Network but restrictions apply to the availability of these data, which were used under license for the current study and so are not publicly available. Data are however available from the authors upon reasonable request and with permission of TriNetX. This study adhered to the STROBE guidelines for observational cohort studies. A completed STROBE checklist is provided in the supplementary materials.

## PREPRINT NOTICE

No prior version of this manuscript has been posted as a preprint.

## Appendix

### Appendix

**Table:** Cohorts Definition Query Criteria for Cohort 1 (query name: Seropositive)

**Ungrouped terms**				
must have		demographics	Age	Age (at least 18 years (most recent occurrence)
cannot have		diagnosis	UMLS:ICD10CM:M06.0	Rheumatoid arthritis without rheumatoid factor
**Group 1 or Group 2 must be present**				
**Group 1**				
**RF group**				
must have		diagnosis	UMLS:ICD10CM:M05	Rheumatoid arthritis with rheumatoid factor
date constraint		The terms in this group occurred between Feb 1, 2015 and Feb 1, 2025		
**Group 2**				
**CCP group**				
must have		procedure	UMLS:CPT:86200	Cyclic citrullinated peptide (CCP), antibody
	and	diagnosis	UMLS:ICD10CM:M05	Rheumatoid arthritis with rheumatoid factor
date constraint		The terms in this group occurred between Feb 1, 2015 and Feb 1, 2025		

This query was run on the Research network, involving 103 healthcare organisations (HCOs). A total of 99 providers responded with patient data. The final cohort included 185,152 patients meeting the query criteria.

**Table:** Query Criteria for Cohort 2 (query name: Seronegative)

**Ungrouped terms**				
must have		demographics	Age	Age (at least 18 years (most recent occurrence))
**Group 1**				
**Seronegative RA**				
must have		diagnosis	UMLS:ICD10CM:M06.0	Rheumatoid arthritis without rheumatoid factor
cannot have		diagnosis	UMLS:ICD10CM:M05	Rheumatoid arthritis with rheumatoid factor
	or	procedure	UMLS:CPT:86200	Cyclic citrullinated peptide (CCP), antibody
date constraint		The terms in this group occurred between Feb 1, 2015 and Feb 1, 2025				

This query was run on the Research network, involving 103 HCOs. A total of 98 providers responded with patient data. The final cohort included 53,364 patients meeting the query criteria.

**Table:** Outcome Definitions

**All-cause mortality**		
**Outcome definition**		
Demographics	Deceased	Deceased
**Settings for the performed analyses**		
Kaplan - Meier survival analysis		excluding patients with outcome prior to the time window
Risk analysis		excluding patients with outcome prior to the time window
**Hospitalisation**		
**Outcome definition**		
Visit	UMLS:HL7V3.0:VisitType:ACUTE	Visit: Inpatient Acute
Visit	UMLS:HL7V3.0:VisitType:IMP	Visit: Inpatient Encounter
Visit	UMLS:HL7V3.0:VisitType:NONAC	Visit: Inpatient Non-acute
Visit	UMLS:HL7V3.0:VisitType:OBSENC	Visit: Observation Encounter
Visit	UMLS:HL7V3.0:VisitType:SS	Visit: Short Stay
Visit	UMLS:HL7V3.0:VisitType:EMER	Visit: Emergency
**Settings for the performed analyses**		
Risk analysis		including patients with outcome prior to the time window
Kaplan - Meier survival analysis		including patients with outcome prior to the time window
**ILD**		
**Outcome definition**		
Diagnosis	UMLS:ICD10CM:J84	Other interstitial pulmonary diseases
Diagnosis	UMLS:ICD10CM:M05.1	Rheumatoid lung disease with rheumatoid arthritis
**Settings for the performed analyses**		
Risk analysis		excluding patients with outcome prior to the time window
Kaplan - Meier survival analysis		excluding patients with outcome prior to the time window
**Pulmonary HTN**		
**Outcome definition**		
Diagnosis	UMLS:ICD10CM:I27.0	Primary pulmonary hypertension
Diagnosis	UMLS:ICD10CM:I27.2	Other secondary pulmonary hypertension
**Settings for the performed analyses**		
Risk analysis		excluding patients with outcome prior to the time window
Kaplan - Meier survival analysis		excluding patients with outcome prior to the time window
**Steroid dependence**		
**Outcome definition**		
Diagnosis	UMLS:ICD10CM:Z79.52	Long term (current) use of systemic steroids
**Settings for the performed analyses**		
Kaplan - Meier survival analysis		including patients with outcome prior to the time window
Risk analysis		including patients with outcome prior to the time window
**DMARDs use**		
**Outcome definition**		
Medication	NLM:RXNORM:1357536	tofacitinib
Medication	NLM:RXNORM:614391	abatacept
Medication	NLM:RXNORM:121191	rituximab
Medication	NLM:RXNORM:612865	tocilizumab
Medication	NLM:RXNORM:1923319	sarilumab
Medication	NLM:RXNORM:819300	golimumab
Medication	NLM:RXNORM:709271	certolizumab pegol
Medication	NLM:RXNORM:327361	adalimumab
Medication	NLM:RXNORM:214555	etanercept
Medication	NLM:RXNORM:191831	infliximab
Medication	NLM:RXNORM:6851	methotrexate
Medication	NLM:RXNORM:27169	leflunomide
Medication	NLM:RXNORM:9524	sulfasalazine
Medication	NLM:RXNORM:5521	hydroxychloroquine
Medication	NLM:RXNORM:1256	azathioprine
**Settings for the performed analyses**		
Risk analysis		excluding patients with outcome prior to the time window
Kaplan - Meier survival analysis		excluding patients with outcome prior to the time window
**RA-related joint damage**		
**Outcome definition**		
Diagnosis	UMLS:ICD10CM:M21.839	Other specified acquired deformities of unspecified forearm
Diagnosis	UMLS:ICD10CM:M20.03	Swan-neck deformity
Diagnosis	UMLS:ICD10CM:M20.02	Boutonnière deformity
Diagnosis	UMLS:ICD10CM:M21.61	Bunion
Diagnosis	UMLS:ICD10CM:M20.5X9	Other deformities of toe(s) (acquired), unspecified foot
Diagnosis	UMLS:ICD10CM:M20.01	Mallet finger
Diagnosis	UMLS:ICD10CM:M20.4	Other hammer toe(s) (acquired)
**Settings for the performed analyses**		
Kaplan - Meier survival analysis		excluding patients with outcome prior to the time window
Risk analysis		excluding patients with outcome prior to the time window
**Chronic CAD**		
**Outcome definition**		
Diagnosis	UMLS:ICD10CM:I25	Chronic ischemic heart disease
**Settings for the performed analyses**		
Risk analysis		excluding patients with outcome prior to the time window
Kaplan - Meier survival analysis		excluding patients with outcome prior to the time window
**ACS**		
**Outcome definition**		
Diagnosis	UMLS:ICD10CM:I24.9	Acute ischemic heart disease, unspecified
Diagnosis	UMLS:ICD10CM:I20.0	Unstable angina
Diagnosis	UMLS:ICD10CM:I21	Acute myocardial infarction
Diagnosis	UMLS:ICD10CM:I22	Subsequent ST elevation (STEMI) and non-ST elevation (NSTEMI) myocardial infarction
**Settings for the performed analyses**		
Kaplan - Meier survival analysis		excluding patients with outcome prior to the time window
Risk analysis		excluding patients with outcome prior to the time window

This table outlines the definitions for each outcome and analysis specifications. Patients with prior occurrences of the outcome before the time window were excluded from specific analyses.

**Table:** Propensity Score Matching

**Cohort 1 and cohort 2 patient count before and after propensity score matching**		
Cohort	Patient count before matching	Patient count after matching

1 - Seropositive	185,153	53,246
2 - Seronegative	53,364	53,246
**Propensity score density function - Before and after matching (cohort 1 - purple, cohort 2 - green)**		

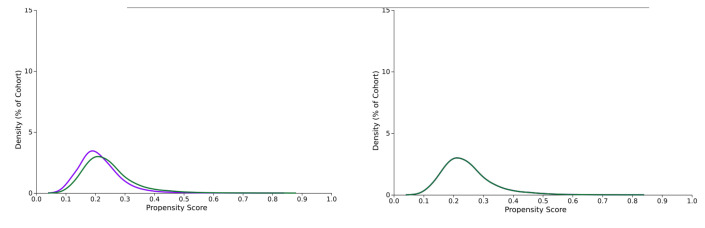

Propensity score matching was performed on all 49 baseline characteristics:

In the Demographics category, patients were matched on Current Age, Age at Index, Female, Male, Black or African American, White, American Indian or Alaska Native, Asian, Native Hawaiian or Other Pacific Islander, Other Race, Unknown Race, Hispanic or Latino, Not Hispanic or Latino, and Unknown Ethnicity characteristics. In the Diagnosis category, patients were matched on Disorders of lipoprotein metabolism and other lipidemias, Essential (primary) hypertension, Nicotine dependence, Personal history of nicotine dependence, Tobacco use, Overweight and obesity, Chronic kidney disease (CKD), Type 2 diabetes mellitus, Other arthritis, Arthropathic psoriasis, Enteropathic arthropathies, Ankylosing spondylitis, Juvenile arthritis, Sacroiliitis, not elsewhere classified, Spinal enthesopathy, Other specified inflammatory spondylopathies, Unspecified inflammatory spondylopathy, and Systemic lupus erythematosus (SLE). In the Medication category, patients were matched on Beta-blocking agents, Angiotensin II inhibitors, ACE inhibitors, Calcium channel blockers, Loop diuretics, Thiazides/related diuretics, Spironolactone, Aspirin, Clopidogrel, Ticagrelor, Prasugrel, Cangrelor, Lipid-modifying agents, Blood glucose-lowering drugs excluding insulins, Insulin, Antidepressants, and Antithyroid agents.
